# Infrared thermography in the assessment of brown and white adipose tissue in children with different nutritional states

**DOI:** 10.1016/j.jped.2025.101498

**Published:** 2026-01-10

**Authors:** Gisele Bailich, Paulo Roberto Santos Lopes, Danubia da Cunha de Sá-Caputo, Alessandro Sartório, Gabriela Peixe, Mario Bernardo Filho, André Everton de Freitas, Anelise Sonza

**Affiliations:** a Universidade do Estado de Santa Catarina, Centro de Ciências da Saúde e do Esporte (UDESC/CEFID), Laboratório de Desenvolvimento e Controle Postural (LADESCOP), Florianópolis, SC, Brazil; b Universidade do Estado de Santa Catarina, Centro de Ciências da Saúde e do Esporte (UDESC/CEFID), Programa de Graduação em Ciências do Movimento Humano, Florianópolis, SC, Brazil; c Universidade do Estado do Rio de Janeiro, Instituto de Biologia Roberto Alcantara Gomes, Laboratório de Vibrações Mecânicas e Práticas Integrativas, Rio de Janeiro, RJ, Brazil; d Istituto Auxologico Italiano, IRCCS Experimental Laboratory for Auxo-endocrinological Research, Piancavallo-Verbania, Italy; e Universidade do Estado de Santa Catarina, Centro de Ciências da Saúde e do Esporte (UDESC/CEFID), Departamento de Fisioterapia, Florianópolis, SC, Brazil

**Keywords:** Adipose tissue, Obesity, Thermography and anthropometry

## Abstract

**Objective:**

To evaluate brown adipose tissue (BAT) and white adipose tissue using Infrared Thermography (IRT) in children with different nutritional statuses and correlate findings with anthropometric variables.

**Methods:**

This cross-sectional observational study assessed body composition via bioelectrical impedance (BIA), skin temperature (supraclavicular and abdominal) through IRT, and anthropometric variables such as neck (NC), waist (WC), and hip (HC) circumferences. Calculations included waist-to-hip ratio (WHR) and conicity index (CI). Statistical analyses were performed in SPSS 20.0, with normality checked by the Shapiro-Wilk and homogeneity by Levene tests. Groups (eutrophic, overweight, obese) were compared using Pearson's Chi-square for categorical variables, Kruskal-Wallis, and ANOVA tests for quantitative data. Correlations were analyzed using Spearman's method. The significance level was set at p ≤ 0.05.

**Results:**

Of 160 participants, 116 children were analyzed (eutrophic: N = 58; overweight: N = 26; obese: N = 32). Significant differences were noted between the eutrophic and obese groups. Supraclavicular temperature negatively correlated with BIA variables (total body water, fat-free mass, body fat percentage).

**Conclusions:**

IRT demonstrated inverse correlations between BAT temperature and BMI, NC, WC, and HC, underscoring its potential in obesity risk analysis. IRT also offers quantitative assessments of BAT activity and may estimate body fat percentages, suggesting its relevance for monitoring adipose tissue function and metabolic effects of physical exercise.

## Introduction

Childhood obesity is a growing global health concern and is associated with early metabolic, cardiovascular, and psychological complications [1]. Adipose tissue plays a central role in energy homeostasis and exists predominantly as white adipose tissue (WAT), specialized in energy storage, and brown adipose tissue (BAT), which dissipates energy through thermogenesis [2]. Because thermogenic activity tends to be higher in childhood[3,4] interest has increased in identifying physiological markers that may help characterize adiposity and metabolic status during this period.

Infrared thermography (IRT) has emerged as a non-invasive, accessible imaging technique capable of capturing regional skin temperature distributions associated with underlying thermogenic processes[5,6] and supraclavicular skin temperature may serve as an indirect indicator of both brown adipose tissue volume and its metabolic activity [7, 8, 9]. Although widely used in animal studies, its application in humans has grown, especially for evaluating supraclavicular BAT-related thermal patterns in adults and children. IRT differs from single-point temperature assessment by producing a two-dimensional thermal image, allowing the analysis of specific anatomical regions such as the supraclavicular area, where BAT may be present, and the abdomen, where WAT predominates [10,11].

It is important to note that the supraclavicular fat depot is anatomically heterogeneous and contains a mixture of brown adipose tissue (BAT), white adipose tissue (WAT), skeletal muscle, and local vasculature. Although BAT is frequently located in this region, it would be inaccurate to assume that the entire supraclavicular volume represents BAT; however, it is expected that BAT will increase local surface temperature in the supraclavicular area in response to the thermogenic effect. Infrared thermography (IRT), therefore, captures the surface temperature resulting from the combined influence of these underlying tissues, rather than the temperature or metabolic activity of BAT in isolation [6,12].

Considering these aspects, the authors hypothesized that supraclavicular and abdominal skin temperatures would differ according to nutritional status and would correlate negatively with measures of adiposity in children. Therefore, the objective of this study was to evaluate BAT- and WAT-related thermal patterns using infrared thermography in children with different nutritional statuses and to examine their correlation with anthropometric variables.

## Materials and methods

### Participants and ethical criteria

This cross-sectional study was conducted from November 2022 to May 2023 in public and private schools located in southern Brazil. A non-probabilistic, intentional sampling method was employed. The inclusion criteria comprised children aged 6 to 12 years of both sexes, regularly enrolled in the school system, and capable of completing the proposed evaluations. Exclusion criteria included chronic illnesses (e.g., cancer, respiratory diseases, neurological syndromes), physical or cognitive disabilities, and inability to perform any part of the assessment protocol.

Prior to data collection, all participants provided assent through a signed Informed Assent Form, and parents or legal guardians signed an Informed Consent Form. The study was approved by the Research Ethics Committee of Santa Catarina State University (approval number: 5.465.762) and followed Resolution 196/96 of the Brazilian National Health Council.

### Anthropometric assessment

Body mass was measured with a calibrated digital scale (Mega Stone®, Advance, China; capacity: 0–180 kg; precision: 50 g), and height was measured using a portable stadiometer (Sanny®, Caprice, Brazil; capacity: 200 cm; precision: 0.1 cm). BMI was calculated as body mass (kg) divided by the square of height (m²), and nutritional status was classified using WHO BMI Z-scores. All assessments were conducted with the children barefoot and dressed in light clothing (shorts and t-shirt), following standardized positioning protocols.

### Body composition analysis (bioelectrical impedance)

Bioelectrical impedance analysis (BIA) was performed using a tetrapolar device (Sanny®, model BIA1010, Brazil). Participants were instructed to abstain from eating for at least 4 h, refrain from physical activity for 24 h, and rest for 10 min prior to the measurement. Metal objects were removed before testing. The following BIA parameters were collected: fat-free mass (FFM, kg), body fat percentage (BF %), and BMI (kg/m²).

### Body circumference measurements

Neck (NC), waist (WC), and hip (HC) circumferences were measured using a non-elastic measuring tape (Sanny®, TR 4013, Brazil; capacity: 150 cm; precision: 0.1 cm), in accordance with the standards of Lohman et al. (1988) and Mussoi (2017). NC was measured at the level of the cricoid cartilage. WC was measured at the midpoint between the last rib and the iliac crest, and HC at the widest point of the gluteal region.

### Infrared thermography protocol

Thermal images were captured using a compact FLIR C3-X thermal camera (USA), equipped with a 128 × 96 infrared sensor (12,288 pixels) and a 5MP optical camera, with a temperature detection range of −20 °C to 300 °C. The camera was mounted on a tripod (Tomate®, model MTG 3018A, Brazil) positioned 1.5 m from the participant and the camera emissivity was 0.98. Children wore swimwear to expose the supraclavicular and abdominal regions. To ensure accuracy, participants rested for 20 min in a thermally controlled room maintained at 21 ± 1 °C before imaging, moderate humidity, avoiding direct air-flow exposure to the air-conditioning. Data collection performed in the morning. The room, door and windows remained closed and away from infrared radiation during all imaging sessions to improve data collection quality. Children were instructed to avoid physical activity, caffeine, large meals, massage or other physical tools that could increase skin temperature for 2 h prior to the test. Participants also avoided any physical contact with the evaluated regions, preventing touch-induced changes in superficial blood flow or skin temperature and the skin was kept clean and dry. Image acquisition was conducted with the subject standing in anatomical position, facing the camera. The use of predefined anatomical regions and a fixed camera-to-subject distance further reduced variability related to the insulation effect of subcutaneous fat. In addition, the standardized resting period and the requirement to stand still in anatomical position helped stabilize cutaneous perfusion before imaging.

### Statistical analysis

Data were analyzed using SPSS version 20.0. Descriptive statistics included mean, standard deviation, median, and interquartile ranges. Normality was assessed via the Shapiro-Wilk test, and homogeneity via Levene’s test. Groups (eutrophic, overweight, obese) were compared using Pearson’s chi-square test for categorical variables, and ANOVA or Kruskal-Wallis tests for quantitative data, depending on distribution. Post hoc analyses included LSD for ANOVA and Dunn-Bonferroni for Kruskal-Wallis. Spearman’s correlation was used for non-parametric variables. A significance level of *p* ≤ 0.05 was adopted.

### Thermal image analysis

**Figure 1:**
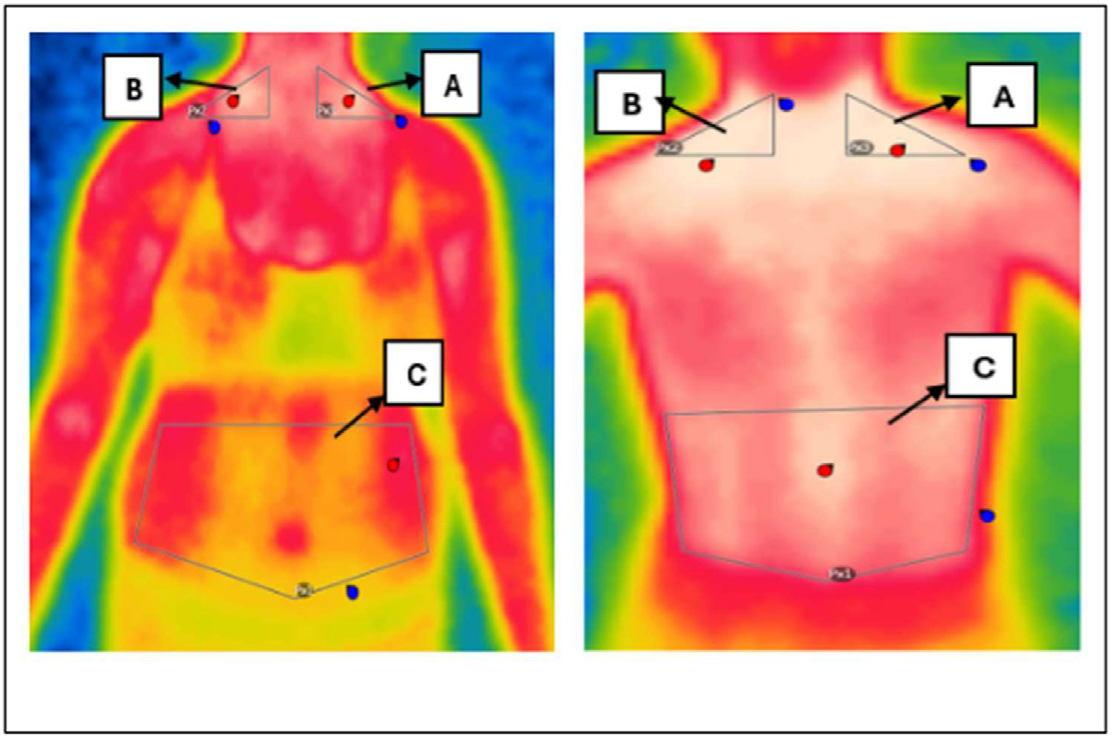
Representation of thermal images taken.

Flir Thermal Studio Pro software was used to interpret the images captured by the thermographic camera. Using this program, Regions of Interest (ROIs) were identified and delimited, allowing skin surface temperatures to be recorded. In each of these ROIs, the software calculated estimates of the minimum, average and maximum temperatures. Triangular areas of interest were positioned in the SCV (Subclavian) region on the left and right sides, as well as a pentagonal-shaped abdominal region, as illustrated in Figure 1.

Representation of thermal images taken in the regions of interest (ROIs), where A: left subclavian area; B: right subclavian area, demarcated by triangles; C: abdominal region, demarcated by a pentagon.

The anatomical positioning of the Regions of Interest (ROIs), including bilateral supraclavicular and abdominal areas, is illustrated in Figure 1.

## Results

**Figure 2:**
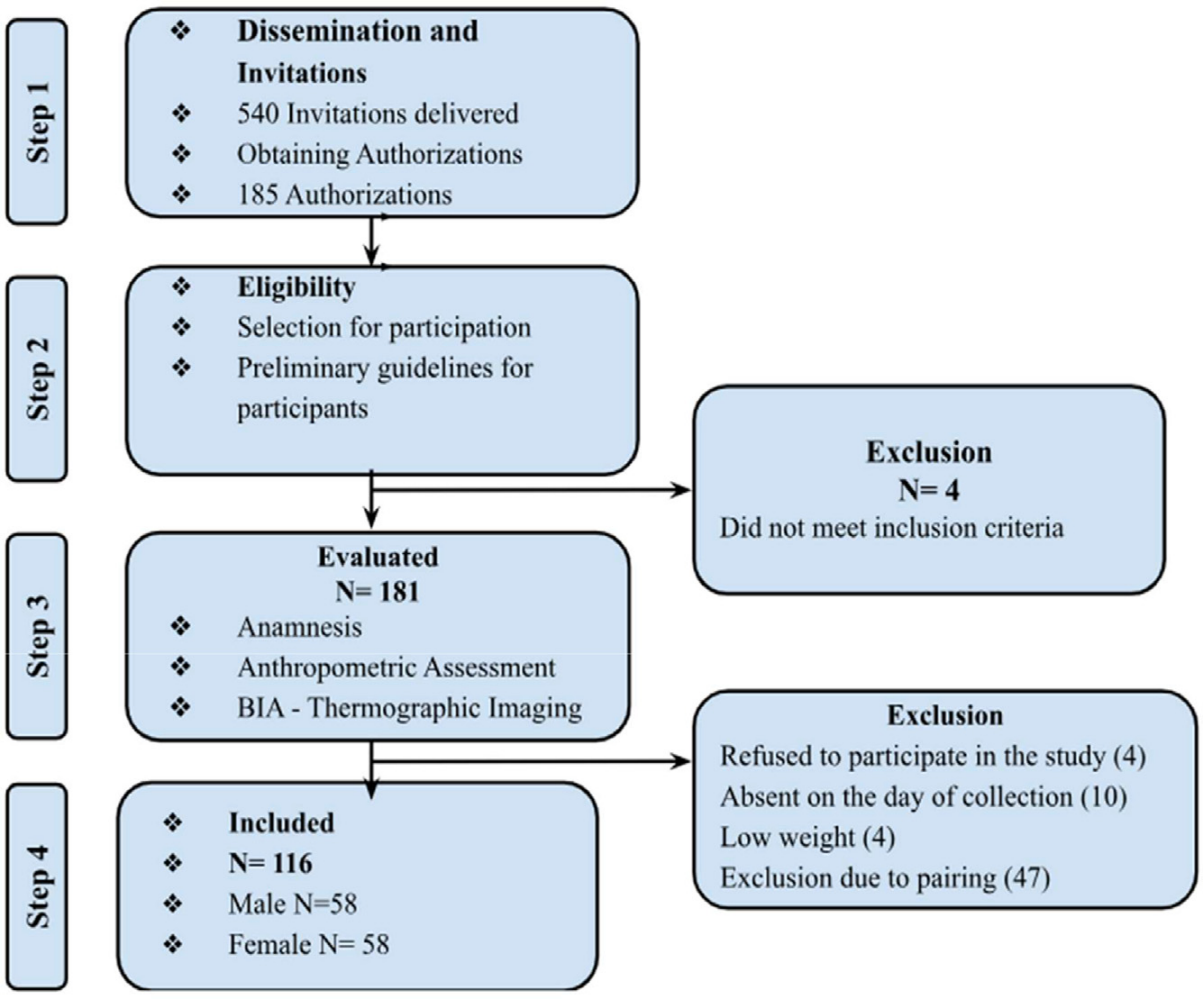
Flowchart of the study's data collection.

According to Figure 2, of the 540 invitations, 181 schoolchildren were assessed, and 116 participants were included in the final analysis after being matched by gender and age with the eutrophic group. A total of 65 students were excluded during this process. The sample consisted of 50 % boys, and most of the sample came from the private school system (80.2 %). Of the participants, 50 % were eutrophic, 22.4 % were overweight, and 27.6 % were obese. This classification was based on an analysis of the BMI Z-score.

BIA, bioelectrical impedance.

**Figure 3:**
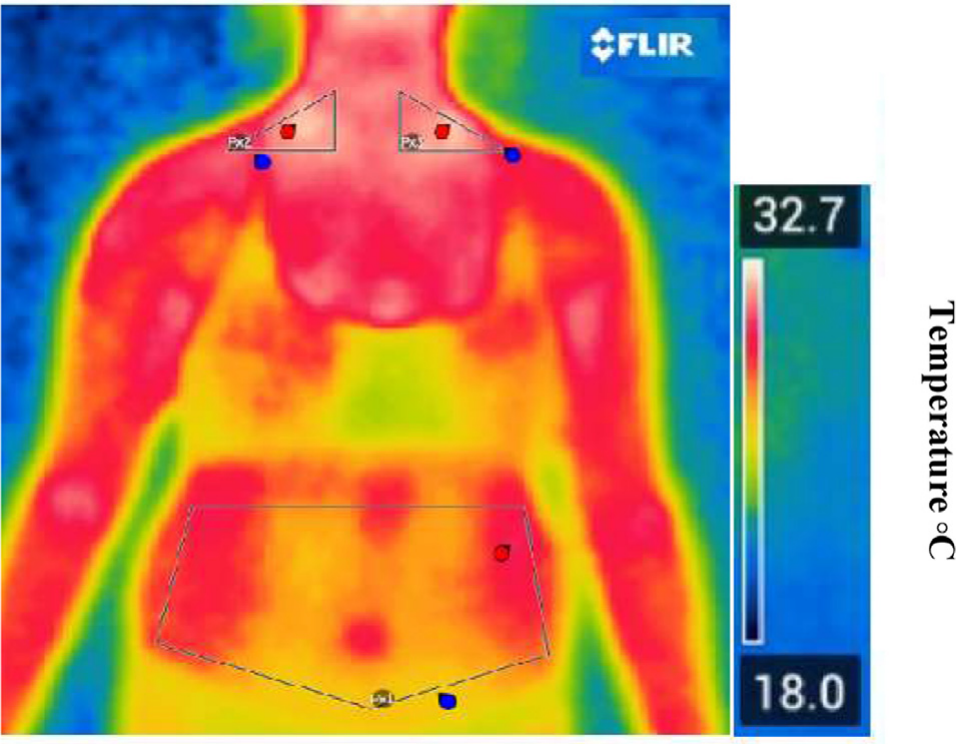
Representative thermal image captured by the thermal camera illustrating temperature distribution across thoracoabdominal regions. The color scale reflects surface temperature, with warmer zones (in red/yellow) indicating increased thermal activity and potential BAT regions.

Figures 1 and 3 present real thermographic images acquired during data collection. The images illustrate the distribution of surface temperature across the thoracoabdominal region, with a corresponding thermal scale to the right of the figure. Warmer colors (red/yellow) indicate higher thermal activity and are associated with regions of potential brown adipose tissue (BAT) activity, particularly in the supraclavicular area. Cooler regions are depicted in shades of blue and green, typically corresponding to areas of lower metabolic activity or subcutaneous white adipose tissue (WAT). This real-life example reinforces the interpretation of thermographic data and the differentiation of body regions based on their thermal profile.

BAT, brown adipose tissue.

Table 1 shows the characterization of the sample, as well as anthropometric variables, nutritional status, the distribution of body composition variables, and skin temperature.

**Table 1:** General characterization of the study sample divided into groups: eutrophic, overweight and obese.

	Nutritional Status Classification				
Variables	Eutrophic	Overweight	Obese	Total	p value
	(*N* = 58)	(*N* = 26)	(*N* = 32)	(*N* = 116)	
**Sex n(%)**					
**Male**	29(50,0)	14(53,8)	15(46,9)	58(50,0)	**0,8702**^2^
**Female**	29(50,0)	12(46,2)	17(53,1)	58(50,0)	
**Age (Years)**	10,0 [7,00; 11,00]	9,0 [7,00; 11,00]	10,0 [8,00; 11,00]	10,00 [7,00; 11,00]	**0,61,81**^1^
**MC (kg)**	30,45 [24,66; 37,33]^a^^b^	39,83 [30,06; 48,03]^c^	51,60 [41,29; 60,43]	36,55 [28,96; 46,89]	**<0,001**^1^
**Height (m)**	1,36 [1,26; 1,47]^b^	1,39 [1,30; 1,52]	1,46 [1,32; 1,56]	1,40 [1,29; 1,50]	**0,03,91**^1^
**BMI (kg/m²)**	16,33 [15,21; 17,90]^a^^b^	19,62 [18,42; 21,30]^c^	24,72 [22,82; 26,26]	18, 51 [16,31; 21,90]	**<0,001**^1^
**Percentile**	52,60 [25,20; 68,17]^a^^b^	90,06 [85,48; 91,22]^c^	97,47 [96,77; 98,24]	83, 37 [52,10; 95, 34]	**<0,001**^1^
**FFM (kg)**	21,30 [18,68; 26,03]^a^^b^	23,97 [21,29; 31,55]	29,46 [23,92; 37,91]	24,06 [20,03; 29,42]	**<0,001**^1^
**BF(%)**	29,62±5,94^a^^b^	33,60±5,54^c^	40,74±6,43	33,14 [27,76; 38,10]	**<0,001**^3^
**NC (cm)**	26,00 [25,00; 28,00]^a^^b^	28,50 [27,00; 30,00]^c^	30,25 [28,63; 32,38]	28,00 [26,00; 29,50]	**<0,001**^1^
**WC (cm)**	56,00 [53,75; 61,13]^a^^b^	63,75 [60,00; 69,75]^c^	77,50 [74,25; 86,00]	63,00 [56,00; 73,75]	**<0,001**^1^
**HQ (cm)**	69,50 [63,00; 77,50]^a^^b^	78,00 [71,00; 84,38]^c^	89,00 [80,25; 95,00]	75,00 [68,25; 85,88]	**<0,001**^1^

Values presented as median and interquartile ranges. MC: Body Mass, BMI: Body Mass Index, FFM: Fat Free Mass, BF ( %) Body Fat Percentage, NC: Neck Circumference, WC: Waist Circumference, HC: Hip Circumference, cm: Centimeters.

1 Kruskal-Wallis test.

2 Asymptotic Pearson's chi-square test

3 Anova test

a Eutrophic different from overweight.

b Eutrophic different from obese.

c Overweight different from obese.

The correlations between nutritional status and infrared thermography variables are shown in Table 2. The data reveal significant differences in temperature measurements between the eutrophic, overweight, and obese groups.

**Table 2:** Classification of the nutritional status with the variables of the infrared thermography.

	Nutritional Status Classification				
Variables (°C)	Eutrophic	Overweight	Obese	Total	p value
	(*N* = 58)	(*N* = 26)	(*N* = 32)	(*N* = 116)	
**RST Mín**	30,55 [29,98; 32,10]^a^^,^^b^	29,30 [28,23; 30,70]	28,95 [26,63; 30,10]	30,15 [28, 43; 31,10]	**<0,001**^1^
**RST Máx.**	32,15 [31,28; 33,35]^a^^,^^b^	31,35 [30,33; 32,10]	30,90 [29,73; 31,78]	31,70 [30,43; 32,48]	**<0,001**^1^
**RST Méd.**	31,50 [30,60; 32,80]^a^^,^^b^	30,55 [29,38; 31,40]	29,95 [28,95; 30,98]	30,90 [29, 63; 31, 78]	**<0,001**^1^
**LST Mín.**	30,15 [29,35; 31,63]^a^^,^^b^	29,05 [28,35; 30,70]	29,00 [26,98; 29,98]	29,80 [28,43; 30,80]	**<0,001**^1^
**LST Máx.**	31,95 [30,83; 33,23]^b^, ^c^	31,20 [30,38; 32,13]	31,00 [29,35; 32,00]	31,65 [30,40; 32,50]	**0,00,71**^1^
**LST Méd.**	31,15 [30,20; 32,50]^b^	30,25 [29,40; 31,45]	30,25 [28,63; 31,20]	28,55 [26,93; 29,70]	**0,00,11**^1^
**AT Mín.**	27,85 [26,73; 29,10]^a^^,^^b^	25,50 [24,03; 26,73]	25,80 [22,93; 27,30]	27,05 [25,03; 28,00]	**<0,001**^1^
**AT Máx.**	30,70 [29,58; 31,93]^a^^,^^b^	29,20 [27,70; 30,20]	29,55 [27,73; 30,68]	30,00 [28,83; 31, 38]	**<0,001**^1^
**AT Méd.**	29,25 [28,33; 30,58]^a^^,^^b^	27,45 [26,00; 28,28]	27,50 [25,05; 29,00]	28,55 [26,93; 29,70]	**<0,001**^1^

Values presented as medians and interquartile ranges. RST, right supraclavicular temperature; LST, left supraclavicular temperature; AT, abdominal temperature; Min, minimum; Max, maximum; Med, median; °C, degrees Celsius.

1 Kruskal–Wallis test.

a Eutrophic different from overweight.

b Eutrophic different from obese.

c Overweight different from obese.

Table 3 shows the correlations between the temperature of brown and white adipose tissue measured by IVT, anthropometric measurements and BIA variables. The results indicate significant negative correlations between the temperatures measured and various anthropometric variables.

**Table 3:** Correlation of brown and white adipose tissue temperature measured by TIV, anthropometric measurements and BIA variables.

Variables °C	NC^1^	WC^1^	HC^1^	FFM(kg)^1^	BF ( %)^1^
**RST Mín**	−0,378^a^	−0,354^a^	−0,354^a^	−0,263^a^	−0,343^a^
**RST Máx.**	−0,343^a^	−0,280^a^	−0,265^a^	−0,212^a^	−0,233^a^
**RST Méd.**	−0,366^a^	−0,319^a^	−0,310^a^	−0,236^a^	−0,290^a^
**LST Mín.**	−0,369^a^	−0,328^a^	−0,297^a^	−0,203^a^	−0,331^a^
**LST Máx.**	−0,270^a^	−0,205^a^	−0,183^a^	−0,119^ns^	−0,219^a^
**LST Méd.**	−0,308^a^	−0,265^a^	−0,233^a^	−0,147^ns^	−0,280^a^
**AT Mín.**	−0,408^a^	−0,371^a^	−0,314^a^	−0,209^a^	−0,336^a^
**AT Máx.**	−0,282^a^	−0,221^a^	−0,179^a^	−0,094^ns^	−0,238^a^
**AT Méd.**	−0,374^a^	−0,330^a^	−0,277^a^	−0174 ns	−0,315^a^

RST, right supraclavicular temperature; LST, left supraclavicular temperature; AT, abdominal temperature; Min, minimum; Max, maximum; Med, mean temperature; NC, neck circumference; WC, waist circumference; HC, hip circumference; FFM, fat-free mass; BF ( %), body fat percentage; °C, degrees Celsius.

1 Spearman’s correlation.

a Indicates statistically significant correlations (*p* ≤ 0.05).

ns Indicates non-significant correlations (*p* > 0.05).

## Discussion

In this study, individuals with higher body mass and BMI showed lower skin temperatures in the assessed regions. Under controlled conditions, skin temperature varies across body areas, being higher over muscular regions compared to bones and tendons [13]. The present findings align with previous research showing an inverse relationship between thermographically estimated BAT activity and both BMI and WC[3], reinforcing the influence of adiposity on thermal responses.

Similarly, a study with 68 children aged 6–11 identified BAT -related “hot spots” influenced by BMI and ethnicity, showing a negative association between BMI and skin temperature [14]. Evidence from adults also indicates that BAT in lean individuals is more readily activated and can be indirectly assessed using IRT [15].

When interpreting these findings, it is important to recognize that supraclavicular temperature reflects the combined influence of multiple tissues rather than BAT alone. Studies in children and adults show that supraclavicular skin temperature is shaped by BAT, subcutaneous WAT thickness, skeletal muscle, and cutaneous perfusion [4,16]. In individuals with greater adiposity, thicker WAT increases thermal insulation, reducing heat dissipation and potentially masking BAT-related heat signals consistent with the lower temperatures observed in children with higher BMI in the present study. Systematic analyses emphasize that IRT measures only surface temperature and therefore reflects integrated regional physiology rather than direct BAT activation [12]. These factors suggest that the temperature differences observed likely arise from the interaction of BAT activity, WAT insulation, and perfusion characteristics, supporting the use of IRT as a complementary indirect marker of thermal physiology in pediatric populations.

Chudecka and Lubkowska demonstrated in adults that IRT-derived skin temperature is associated with body fat percentage estimated by BIA, highlighting consistent thermal differences across multiple body regions in both men and women [17,18]. In the comparison between thermographic variables and BIA, body fat percentage correlated with all thermal measurements, whereas lean mass showed stronger associations with supraclavicular temperatures, which are partially influenced by BAT. Although statistically significant, most correlations were weak to moderate and negative, reinforcing that the relationship between skin temperature, adiposity, and lean mass is multifactorial and cannot be explained by a single physiological component.

A study of Brazilian Army soldiers (25–35 years) found negative correlations between lean mass and skin temperature across multiple body regions, reinforcing that individuals with greater subcutaneous fat show lower surface temperatures due to reduced heat dissipation compared with lean tissue [12,19]. Although these findings support the insulating effect of adiposity, there remains a notable gap regarding how this relationship manifests in pediatric populations, particularly in the context of childhood obesity.

Large-scale evidence also underscores the metabolic relevance of BAT. In a cohort of 52,000 adults, BAT presence was associated with markedly lower prevalence of cardiometabolic diseases, including type 2 diabetes, dyslipidemia and hypertension, with the strongest protective effects observed in individuals with overweight or obesity [20]. These findings highlight the potential of BAT to mitigate the adverse metabolic consequences of excess adiposity.

In line with these findings, specific body areas show associations between adipose tissue and skin temperature. Previous research demonstrated that BAT activation is negatively correlated with BMI and other anthropometric variables, which is relevant for the early identification of excess body mass in children, especially given the limitations of BMI for assessing body composition [3].

In this context, IRT can support the assessment of body fat distribution when used alongside other anthropometric indicators [13]. Beyond identifying adiposity, several studies have attempted to link anthropometric measures with body composition assessed by BIA [21,22]. Although BMI is widely applied to estimate body fat, it has clear limitations, particularly in pediatric populations [23].

A study reported a significant disparity in the discriminative capacity of BMI and BIA across different body composition categories. BIA proved to be more accurate for assessing obesity in children due to its direct estimation of body fat and its ability to distinguish between fat and lean mass [24]. Although DEXA remains the gold standard, its clinical use is limited by cost and availability.

According to Leitner et al., body fat distribution is a major risk factor for coronary artery disease. Epidemiological evidence consistently shows the relevance of visceral fat in the development of metabolic and cardiovascular conditions in both children and adults [10,20]. Therefore, anthropometric methods remain essential for preventive and interventional strategies aimed at improving metabolic and cardiovascular.

Recently, IRT has been proposed as an alternative approach to estimate BAT activity because it is accessible, fast, non-invasive, and safe for pediatric applications. Its feasibility is supported by the superficial anatomical location of supraclavicular BAT, which lies close to the skin surface and therefore contributes to regional thermal patterns detectable by IRT [12].

A systematic review highlighted significant advances in the use of IRT to estimate BAT activity, although there is still a lack of additional studies to validate this technique. Few studies have proposed cut-off points for classifying BAT activity [4, 5, 6,25].

Given BAT thermogenic capacity, IRT presents itself as a promising tool not only to detect BAT, but also to investigate its function [6]. In many studies that used IRT to measure BAT, participants were subjected to cold-induced conditions or received thermogenic substances, such as caffeine and capsinoids, and pharmacological agents. In all these studies, adipose tissue activation was identified using IRT [6,15,25, 26, 27, 28, 29].

This study represents a pioneering initiative in the use of IRT for the detection of BAT and WAT in pediatric individuals. The present analysis indicates that IRT is a promising, non-invasive technique for identifying BAT in the supraclavicular region. The discriminant measure used is the difference in skin temperature between the region of interest after acclimatization for thermographic image capture. The authors observed that this temperature difference has a better predictive value from right to left, contrasting with the results of a study conducted in adults, where they had a higher temperature on the left than on the right [28]. Therefore, IRT proves to be a valuable technology that can complement other anthropometric variables in the study of BAT and obesity.

Although the authors observed a negative association between skin temperature and adiposity at the population level, interpreting IRT at an individual level requires caution. The supraclavicular region is anatomically heterogeneous and composed not only of BAT, but also of WAT, skeletal muscle, and local vasculature; therefore, it is inaccurate to assume that the entire depot represents BAT. Under thermoneutral conditions, the supraclavicular temperature reflects the integrated influence of BAT activity, subcutaneous WAT thickness, and cutaneous perfusion. Because the supraclavicular region contains a mixture of BAT, WAT, skeletal muscle, and vasculature, the surface temperature measured by IRT reflects the combined influence of these tissues rather than BAT activity alone. In children with higher adiposity, the thicker subcutaneous fat layer increases thermal insulation and attenuates heat dissipation, leading to lower supraclavicular temperatures [12].

Evidence from thermographic and imaging studies supports this interpretation: previous work demonstrated a strong negative correlation between supraclavicular skin temperature and subcutaneous fat thickness, reinforcing that adiposity markedly affects thermal signals obtained under thermoneutral conditions. These mechanisms together help explain the lower temperatures observed in children with elevated BMI in our study and highlight that IRT captures integrated regional physiology rather than isolated BAT activation [30].

For this reason, the authors do not propose IRT as a stand-alone method for estimating BAT activity or body composition. Instead, it should be viewed as a complementary physiological marker that reflects regional heat loss and tissue interactions beyond what is captured by anthropometry or bioimpedance. In children, who tend to have more active BAT depots and thinner subcutaneous layers, IRT may provide additional metabolic insights that extend beyond structural composition measures.

Importantly, our findings do not suggest that IRT should replace established methods such as anthropometry or BIA. These techniques quantify body size and tissue proportions, whereas IRT captures functional thermal responses related to regional metabolic activity. Therefore, IRT provides an additional, physiologically informative dimension of analysis, particularly in pediatric populations, by detecting thermal patterns not accessible through conventional body composition assessments. Its value lies in complementing, rather than substituting, standard evaluation methods.

A limitation of IRT is its ability to evaluate only superficial thermal signals, which restricts its usefulness for studying deeper BAT depots, particularly in the supraclavicular region^6^. Moreover, the scarcity of pediatric studies across different ages and nutritional conditions limits the broader understanding of BAT development during childhood.

In line with the hypothesis established, it was observed that IRT appears to be an effective tool for assessing adipose tissue in different nutritional states. The results showed that body composition, body fat percentage, and muscle mass significantly influence skin temperature measured by IRT. Eutrophic individuals had higher skin temperatures, showing the impact of nutritional status on temperature measurements. These findings suggest that IRT can provide valuable information on the distribution and activity of adipose tissue, contributing to a deeper understanding of the metabolic variations associated with different nutritional conditions.

The study highlights the need for further research involving the pediatric population to validate and standardize the use of IRT in assessing BAT. Although IRT has shown potential in predicting body composition in schoolchildren, further studies with larger samples are needed, especially since most current research uses cold exposure methods to activate the TAB. Studies applying IRT in pediatric individuals are still limited, underlining the importance of future research. In addition, the study emphasizes the relevance of associating IRT with BIA analysis and other anthropometric variables as effective tools for assessing body fat in pediatric populations, highlighting its practical and clinical importance.

## Funding

This study was financed in part by the Coordenação de Aperfeiçoamento de Pessoal de Nível Superior - Brasil (CAPES) - Finance Code 001 and Fundação de Amparo à Pesquisa e Inovação do Estado de Santa Catarina (Fapesc) FAPESC 2023TR000594.

## Consent to participate

Written informed consent was obtained from the parents.

## Consent to publish

The authors affirm that the human research participants provided ongoing informed consent throughout the research for the publication of the thermal images in Figures 1 and 3.

## Declaration of generative AI in scientific writing

During the preparation of this work, the author(s) used Linguee to assist with the translation process. After using this tool/service, the author(s) reviewed and submitted the manuscript to a paid company (Editage) for the English language review process. The authors edited the content as needed and take full responsibility for the content of the published article.

## Data availability

The data that support the findings of this study are available from the corresponding author.

## Conflicts of interest

The authors declare no conflicts of interest.
